# 

**Published:** 1924-10

**Authors:** 


### THE Hospital and Health REVIEW

A JOURNAL FOR EVERYONE INTERESTED IN PUBLIC HEALTH, PREVENTIVE AND CURATIVE MEDICINE AND HOSPITAL AND INSTITUTIONAL WORK.

*Established 1886.*

Published MONTHLY, Price 6d.

THE HOSPITAL AND HEALTH REVIEW includes within its sphere the various aspects of PUBLIC AND PERSONAL HEALTH, PREVENTIVE AND CURATIVE MEDICINE, HOSPITAL AND INSTITUTION WORK, AND THE GENERAL WELL-BEING OF THE PEOPLE. These subjects of truly vital concern are dealt with in plain, and as far as possible non-technical, language, so that they may be understood, and consequently of *service to everyone* interested in these matters.

Price 6d. of all Booksellers and Newsagents, or 7d. post free of the Manager, "The Hospital and Health Review," 28/29 Southampton Street, Strand, London, W.C. 2.

### THE FARR FUND.

THIS fund, which has been steadily increasing, now totals £129 5s. 0d. The President hopes to raise this to £150. There must still be members of the Society who have not yet contributed, but will like to do so. If they would send in their subscriptions at an early date it would help materially, as to be of real good the fund cannot be kept open much longer.

### The Library of the Bristol Medico-Chirurgical Society.

*The following donations have been received since the publication of the List in July,* 1924.

*October,* 1924.

| | | |
|---|---|---|
| Dental Board of the United Kingdom (1) | .. | 1 volume. |
| Mr. Hey Groves (2) | .. .. .. .. | 1 ,, |
| Medical Research Council (3) | .. .. .. | 2 volumes. |
| Surgeon-General of the United States Army (4) | | 2 ,, |

Unbound periodicals have been received from Lieut.-Col. A. B. Prowse, M.D., F.R.C.S., and Mr. Hey Groves.

### THE ONE HUNDRED AND TENTH LIST OF BOOKS.

| | | | |
|---|---|---|---|
| **Buchanan, A.** | ..*Midwifery Mechanics* .. .. .. .. .. .. .. .. | | 1924 |
| **Burridge, H. A.** | ..*An Introduction to Forensic Medicine* .. .. | | 1924 |
| *Child Life Investigations* | .. .. .. .. .. .. .. .. .. .. .. .. | (3) | 1924 |
| **Culpin, M.** | .. ..*The Nervous Patient* .. .. .. .. .. .. .. .. | | 1924 |
| **Fitzwilliam, D. C. L.** | *On the Breast* .. .. .. .. .. .. .. .. .. .. | | 1924 |
| *Growth of the Jaws, Normal and Abnormal, in Health and Disease* (Five Lectures) | .. .. .. .. .. .. .. .. | (1) | 1924 |

**Haig, H. A.** .. .. *An Introduction to the Histology of Tumours* 1924
**Hurst, A. F.** .. .. *Essays and Addresses on Digestive and Nervous Diseases, etc.* .. .. .. .. .. .. .. .. 1924
*Index-Catalogue, Library of the Surgeon-General's Office, United States Army.* .. 3rd Ser., Vol. IV. (4) 1923
**McDonagh, J. E. R.** *The Nature of Disease* .. .. .. .. .. .. 1924
**Martindale and Westcott.** *The Extra Pharmacopœia.* 18th Ed. Vol. I. .. .. .. .. .. .. .. .. .. .. 1924
*Medical Department of the United States Army in the World War,* Vol. XI. .. .. .. .. .. .. .. (4) 1924
**Muir, R.** .. .. *Text-Book of Pathology* .. .. .. .. .. .. 1924
**Oehlecker, F.** .. *Tuberkulose der Knochen und Gelenke* .. (2) 1924
**Pickerill, H. P.** .. *Facial Surgery* .. .. .. .. .. .. .. .. .. 1924
*Report on the Nutrition of Miners and their Families* .. .. (3) 1924
**Schmidt, P.** .. .. *The Theory and Practice of the Steinach Operation* .. .. .. .. .. .. .. .. .. 1924

TRANSACTIONS, REPORTS, JOURNALS, ETC.

American Ophthalmological Society, Transactions of the Vol. XXI. 1923
College of Physicians of Philadelphia, Transactions of the, 3rd Ser. Vol. XLV. 1923
Medical Officer of Health, Bristol, Annual Report of the .. .. 1923
University of Otago Medical School, Proceedings of the, No. 2 .. 1924

### Local Medical Notes.

MARRIAGE.—On August 18th, at Crantock, Cornwall, the marriage took place of J. A. Nixon, C.M.G., M.D., F.R.C.P., Professor of Medicine, University of Bristol, and Physician, Bristol Royal Infirmary, to Miss D. G. C. Walker, M.R.C.S., L.R.C.P.

**Postgraduate Demonstrations of the University of Bristol** are to be held at the Town Club, 59 Bath Road, Swindon, on Wednesdays, at 3.15 p.m., viz.: October 29th, "Hepatic Insufficiency, Diagnosis, etc." (Dr. A. T. Todd); November 5th, "Inter-relations of Internal Secretion Glands" (Professor F. H. Edgeworth, M.D.); November 12th, "Tonsils and Adenoids and Ear Discharges" (Dr. A. J. Wright); November 19th, "Care and Treatment of Children's Mouths" (Mr. L. E. Claremont, M.R.C.S., L.D.S.); November 26th, "Defective Dietary as a Cause of Children's Disorders" (Dr. O. C. M. Davis); December 3rd, "Dyspepsia: some considerations for diagnosis and treatment" (Dr. Carey Coombs).

Dr. F. C. V. Thompson, Wroughton, Swindon, is the acting Hon. Sec. The fee is Two Guineas for the course.

### *PUBLICATIONS RECEIVED.*

From Edward Arnold & Company, London:

*Text-book of Pathology.* By Robert Muir, M.A., M.D., Sc.D., F.R.S. 1924. Price 35s. net.

From William Blackwood & Sons, Edinburgh:

*Practical Nursing, including Hygiene and Dietetics.* By the late Herbert E. Cuff, M.D., F.R.C.S. and W. T. Gordon Pugh, M.D., B.S. Sixth Edition. 1924. Price 10s. 6d. net.

From G. W. Carnrick Company, New York:

*Organotherapy in General Practice.*

From Government Printing House, Washington:

*Index-catalogue of the Library of the Surgeon-General's Office, United States Army.* Third Series. Vol. IV., 1923.

From William Heinemann, London:

*The Theory and Practice of the Steinach Operation.* By Dr. Peter Schmidt (Berlin). 1924. Price 7s. 6d. net.

*Essays and Addresses on Digestive and Nervous Diseases and on Addison's Anæmia and Asthma.* By Arthur F. Hurst, M.A., M.D., F.R.C.P. 1924. Price 21s. net.

*The Nature of Disease.* By J. E. R. McDonagh, F.R.C.S. 1924. Price £3 3s. od. net.

*On the Breast.* By Duncan C. L. Fitzwilliams, C.M.G., M.D., Ch.M., F.R.C.S. 1924. Price 30s. net.

From Henry Kimpton, London:

*An Introduction to the Histology of Tumours.* By Harold A. Haig, M.B., B.S., D.P.H., M.R.C.S., L.R.C.P. 1924. Price 7s. 6d. net.

From H. K. Lewis & Company, London:

*The Extra Pharmacopœia.* Vol. I. 18th Edition. Revised by W. H. Martindale, Ph.D., F.C.S., and W. Westcott, M.B., D.P.H. 1924. Price 27s. 6d. net.

*An Introduction to Forensic Medicine.* By H. A. Burridge, M.A., M.B. 1924. Price 10s. 6d. net.

*The Nervous Patient.* By Millais Culpin, M.D., F.R.C.S. 1924. Price 10s. 6d. net.

From E. & S. Livingstone, Edinburgh:

*Facial Surgery.* By H. P. Pickerill, C.B.E., M.D., M.S. 1924. Price 21s. net.

*Handbook of Skin Diseases.* By Frederick Gardiner, M.D., B.Sc. (Public Health), F.R.C.S.E., F.R.S.E. Second Edition. 1924. Price 10s. 6d. net.

From Humphrey Milford, Oxford University Press:

*Midwifery Mechanics.* By Lieut.-Col. Andrew Buchanan, I.M.S. (Retired), M.A., M.D., M.Ch., M.A.C. 1924. Price 7s. 6d. net.

From Wakley & Sons, London:

*Guy's Hospital Reports.* July, 1924. Annual Subscription £2 2s. od. net.

### INDEX.

Ackland, W. R., Grave Cardiac Symptoms due to Multiple Dental Abscess, 79.

Association of Physicians of Great Britain and Ireland, 153.

Auricular Failure.—Carey F. Coombs and C. E. K. Herapath, 180.

Board, E. C., Obituary, 157.

**Books, Reviews of—**

Allan, G. A.—Defects in Cardiac Rhythm, 145.

Allbutt, Rt. Hon. Sir T. C.—Notes on Composition of Scientific Papers, 211.

Angina Pectoris.—Sir J. Mackenzie, 34.

Aromatics and the Soul.—D. Mackenzie, 40

Asthma.—F. Coke, 83.

Bayley, W. Wansey.—Venereal Disease, 211.

Cancer, theories and problems of—C. E. Walker, 38.

Capparoni, P. — "Magistri Salernitani nondum Cogniti," 84.

Cardiac Rhythm, defects in.—G. A. Allan, 145.

Chronologia Medica.—Sir D'Arcy Power, 84.

Coke, F.—Asthma, 83.

Common Infections of the Female Urethra and Cervix.—F. Kidd and A. M. Simpson, 144.

Diseases of the Ear.—W. Milligan and W. Wingrave, 38.

Ear, Nose and Throat Treatment.—G. Portmann, 146.

Electric Ionization.—A. R. Friel, 39.

Elements of Surgical Diagnosis.—Sir A. Pearce Gould, 34.

Emergency Operations.—H. C. Orrin, 144.

Fractures, treatment of—C. Max Page and W. R. Bristow, 147.

Friel, R.—Electric Ionization, 39.

Gastric & Duodenal Ulcers.—Sir Berkeley Moynihan, 143.

Golden Rules of Dental Mechanics.—H. Osborn, 83.

Gonorrhœa.—D. Thomson, 211.

Gould, Sir A. Pearce.—Elements of Surgical Diagnosis, 34.

Halls-Dally, J. F.—High Blood Pressure, 143.

Ham, E. B.—A Synoptic Chart of Skin Diseases, 142.

Hawkins, E.—Medical Climatology of England and Wales, 142.

Hayem, G. L'Hématoblaste, troisième élément du sang, 36.

**Books, Reviews of** (*continued*)—
Heart Disease, modern methods in diagnosis and treatment. — F. Heatherley, 83.
Heatherley, F.—Heart Disease, 83.
Hehir, Sir P.—The Medical Profession in India, 39.
Hématoblaste, L', troisième élément du sang.—G. Hayem, 36.
High Blood Pressure.—J. F. Halls-Dally, 143.
Hygiene and Public Health.—Parkes and Kenwood, 35.
Irwin, W. K.—An Introduction to Surgica Urology, 86.
Jack, W. R.—Wheeler's Handbook of Medicine, 145.
Jones, W. Carmalt.—Organic Substances: Sera and Vaccines, 210.
Kidd, F. and Simpson, A. M.—Common infections of the Female Urethra and Cervix, 144.
Lockhart-Mummery, C.—Diseases of the Rectum and Colon, 85.
Mackenzie, Sir J.—Angina Pectoris, 34.
McKenzie, D.—Aromatics and the Soul, 40.
" Magistri Salernitani nondum Cogniti."—P. Capparoni, 84.
Male Organs of Generation, diseases of—K. M. Walker, 85.
Mann, M.—Text-book of Tracheo-Bronchoscopy, 141.
Medical Climatology of England and Wales—E. Hawkins, 142.
Medical Profession in India.—Sir Patrick Hehir, 39.
Milligan, W., and Wingrave, W.—Diseases of the Ear, 38.
Moynihan, Sir B.—Gastric and Duodenal Ulcers, 143.
Nose, Throat and Ear, Diseases of—A. Logan Turner and others, 146.
Oral Hygiene.—J. Sim Wallace, 82.
Organic Substances, Sera and Vaccines.—D. W. Carmalt Jones, 210.
Orrin, H. C.—Emergency operations, 144.
Osborn, H.—Golden Rules of Dental Mechanics, 83.
Page, C. Max, and Bristow, W. R.—The treatment of fractures, 147.
Parkes, L. C. and Kenwood, H. R.—Hygiene and Public Health, 35.
Pharmacology and Therapeutics, a text-book of—E. Poulsson, 35.
Portmann, G.—Ear, Nose and Throat Treatment, 146.
Poulsson, E.—A Text-book of Pharmacology and Therapeutics, 35.
Power, Sir D'Arcy.—Chronologia Medica, 84.

**Books, Reviews of** (*continued*)—
Prescriptions, incompatibility in. — T. Stephenson, 86.
Rectum and Colon, diseases of—Lockhart-Mummery, C., 85.
Scientific Papers, notes on composition of—Rt. Hon. Sir T. Clifford Allbutt, 211.
Skin Diseases, Synoptic Chart of—E. Burnett Ham, 142.
Stephenson, T.—Incompatibility in Prescriptions, 86.
Surgical Operations, hand-book of—Sir F. Treves, 145.
Surgical Urology.—W. K. Irwin, 86.
Thomson, D.—Gonorrhœa, 211.
Tracheo-Bronchoscopy, text-book of—M. Mann, 141.
Treves, Sir F.—Students' hand-book of Surgical Operations, 145.
Turner, A. Logan and others.—Diseases of Nose, Throat and Ear, 146.
Venereal Diseases.—W. Bayley, 211.
Walker, C. E.—Theories and Problems of Cancer, 38.
Walker, K. M.—Diseases of the Male Organs of Generation, 85.
Wallace, J. Sim.—Oral Hygiene, 82.
Wheeler's Handbook of Medicine.—W. R. Jack, 145.

Bristol Medico-Chirurgical Society, 42, 156.
Bristol "Milk Week," 41.

Cancer of the Breast.—A. R. Short, 64.
Cardiac Symptoms.—W.R. Ackland, 79.
Carwardine, T.—Duodenal Ulcers, 16.
Colston Research Society, 215.
Coombs, Carey F. and Herapath, C. E. K.—Auricular Failure, 180.
Crippled Children's Claims, 151.
Critchley, Macdonald.—The Parkinsonian Syndrome, 25.

Diverticulitis of the Pelvic Colon.—Sir C. Gordon-Watson, 112.

Farr, F. G.—Obituary, 155, 158.
Firth, J. L.—Evolutionary History of Renal Surgery, 2, 49.
Forbes Fraser Memorial Hospital, 154.
Forbes Fraser, Obituary, 159.

Gallstone through the Intestine, obstruction due to passage of—C. A. Morton, 126.
General Surgery, a few suggestions in—C. F. Walters, 202.
Gordon-Watson, Sir C.—Diverticulitis of the Pelvic Colon, 112.
Griffiths, L. M.—In Memoriam, 213.

Harrison, A. J.—Obituary, 87, 93.

Herapath, C. E. K. and Coombs, Carey F.—Auricular Failure, 180

Hetling, Henry E.—Obituary, 47.

Hospital Problem of the Labour Party, The, 148.

Labyrinthitis—E.Watson-Williams, 135.

Lansdown, R. G. Poole.—Obituary, 47, 87, 89, 155.

Leprosy problem, The, and its bearing on Tuberculosis.—Sir L. Rogers, 19.

Library of the Bristol Medico-Chirurgical Society, 45, 162, 214, 215.

Local Medical Notes, 48, 95, 163, 216.

Long Fox Lecture: The Conditioned Response.—C. Lloyd Morgan, 166.

Morgan, C. Lloyd.—Long Fox Lecture: The Conditioned Response, 166.

Morton, C. A.—Obstruction due to the passage of a Gallstone, 126.

Parkinsonian Syndrome, The—Macdonald Critchley, 25.

Pinch, A. E. Hayward.—The Therapeutic uses of Radium, 97.

Radium, the Therapeutic uses of—A. E. H. Pinch, 97.

Renal Surgery, Evolutionary History of—J. L. Firth, 2, 49.

Rogers, Sir Leonard.—The Leprosy Problem and its bearing on Tuberculosis, 19, 41.

Short, A. Rendle.—Cancer of the Breast, 64.

Walters, C. F.—A few suggestions in General Surgery, 202.

Watson-Williams, E.—Labyrinthitis, 135.

